# Development of basic-needs experience and finance-related attitude through game-based learning: The role of game mechanics and debriefing

**DOI:** 10.1371/journal.pone.0337686

**Published:** 2025-12-02

**Authors:** Liane Platz, Michael Juettler

**Affiliations:** 1 Laboratory Dr. Liane Platz, Department of Economics, University of Konstanz, Konstanz, Germany; 2 Center for Innovative Teaching and Learning, School of Management and Law, Zurich University of Applied Sciences (ZHAW), Winterthur, Switzerland; Universiti Tenaga Nasional, MALAYSIA

## Abstract

Game-based learning (GBL) supports the development of domain-specific competencies, especially knowledge, more effectively than traditional learning methods. However, it is still unclear which elements of GBL positively impact learning outcomes such as motivation and attitudes. This study involved 293 upper-secondary students in a quasi-experiment using a 2 × 2 control group design with five measurement points to test the effects of game mechanics and reflection prompts. This approach aimed to assess their effects on the basic needs experience and attitude towards financial planning among upper-secondary school students. We applied a multivariate analysis of variance and a moderated mediation path model to address two questions: (a) whether game experiences differ depending on core mechanics and reflection prompt design, and (b) whether game mechanics influence changes in finance-related attitude through basic needs experience, moderated by reflection prompts. The findings demonstrate that games offering a higher degree of strategic decision-making options and direct reflection prompts positively impact students’ basic needs experience with a moderate effect size. However, the path model did not reveal a group-specific influence on finance-related attitude. Therefore, this study provides initial results from an experimental approach to identifying the influence of different game mechanics and reflection prompts, offering valuable empirical insights into the implementation of GBL in educational settings across domains.

## 1 Introduction

The application of game-based learning methods (GBL) is becoming increasingly popular. Such approaches have a long tradition in primary education [1], but GBL is also being implemented and studied more frequently in secondary and tertiary education [2]. This is not only due to the high level of individual interest in gaming among adolescents and young adults but also to its potential based on learning theory that extends comprehensively to the areas of cognition, behavior, and motivation (for an overview, see [3]).

GBL approaches are also applied to promote financial literacy, to simulate financial decision-making in a failure culture [4,5]. In a world with a growing risk shift toward the individual, liberalized financial markets and increasingly complex financial products, a competent and reflective dealing with financial matters is becoming more important [6]. School-based support enables proactive financial education by influencing young people before major financial decisions [7,8], with attitudes serving as key predictors of future financial behavior [7,9].

The main theoretically assumed advantage of GBL across domains relates to motivation, particularly based on Deci and Ryan’s self-determination theory [3]. Empirically, however, a motivational advantage can only be confirmed to a limited extent in the context of reviews but not across domains [10,11] or in economics education [12]. In addition to different research approaches, another reason for divergent findings is the diverse design of GBL, which means that the effect of GBL can only be analyzed to a limited extent in the comparison of learning media [13]. Instead, more precise insight into the effectiveness of GBL can be gained through design comparisons—that is, through the systematic variation of building blocks within the framework of GBL [cf. 14].

Building on the input–process–output model of serious game design by Garris et al. [15], we conducted a multivariate analysis of variance and a moderated mediation path model using data from an intervention study in upper secondary school (n = 293). The analyses addressed two research questions: (RQ1) How does the game experience in a serious game differ depending on variations in core game mechanics and reflection prompts? and (RQ2) How do game mechanics influence finance-related attitude change through the mediation of game experience and the moderation of reflection prompts? To the best of our knowledge, no intervention study to date has systematically examined the combined effects of the primary game mechanic—which determines students’ actions during play—and reflection prompts designed to encourage metacognitive engagement with those actions, while simultaneously providing a robust theoretical foundation for the approach.

## 2 Theoretical background

### 2.1 Financial literacy and the role of finance-related attitudes

#### 2.1.1 Financial literacy.

Financial literacy is defined as financial competence based on the Organisation for Economic Cooperation and Development (OECD)’s framework model for financial literacy for young people in Europe aged 16–18 [16]. The framework specifies four key content areas: (1) money and transactions, (2) planning and managing finances, (3) risks and rewards, and (4) financial landscape. These areas are further categorized into three competence domains: (a) knowledge, (b) behaviors, and (c) attitudes, each of which is associated with specific competency objectives (see [16]). The framework adopts a competence-oriented approach that incorporates volitional and motivational aspects, offering a multi-perspective approach to content that extends beyond consumer education. It also integrates an economic–civic dimension through the inclusion of the financial landscape [17–19].

Empirical findings indicate that financial literacy is positively correlated with stock market participation and fewer financial difficulties, thereby enhancing financial well-being [20,21]. Financial literacy levels among high school students are notably low [21]. The findings on the level of financial literacy with regard to the finance-related knowledge of young people show that, regardless of the type of school, they perform less well in areas in which they have little experience [22,23]. Given that competent handling of financial matters is a lifelong endeavor, the primary goal of school-based interventions is not merely to immediately deliver the required knowledge and skills but rather to adopt a proactive approach [7] in preparing students for lifelong engagement with financial topics. Established (learning) psychological theories [24] point out that competence facets, such as attitudes, social norms, and control beliefs, are essential for behavioral changes and that their promotion should be increasingly taken into account in financial education programs because they can act as mediators between knowledge and behavior [25]. Therefore, the motivational aspects of financial literacy (e.g., attitudes) should be addressed as a certain belief lens that influences financial decisions and downstream behavior (see [6,9,21]).

#### 2.1.2 Why finance-related attitudes matter.

Researcher largely agree on the conceptualization of attitude in terms of its evaluative character [26–28]. Attitudes are therefore highly correlated with values such as basic needs: If individual values are taken into account in an intervention, this can lead to a greater change in attitude [29], for example, by increasing the experience of autonomy in decision-making processes.

This is also evident at the content level, where Barry identified five finance-related facets that illustrate the content structures of attitudes: (1) prestige/power, (2) financial planning, (3) quality, (4) significance, and (5) attachment to money [30]. In the context of financial education, promoting a positive attitude toward financial planning is consistent with the overarching goal of encouraging responsible financial behavior. In contrast, promoting a positive attitude toward the other dimensions carries the risk of a paternalistic attitude, as such efforts may not take sufficient account of the heterogeneity of lifestyles and value systems [31].

Attitudes can be formed through experiences based on socialization processes, and the changeability of attitudes can be traced back to the extent to which they are partly anchored in memory and partly constructed on the fly [28]. There are indications that attitudes show a higher degree of changeability with less experience in finance [30], although evidence on finance-related attitudes is still limited [32,33].

### 2.2 Game-based learning to promote financial literacy

Reviews on financial education confirm the effectiveness of intervention programs in terms of subject knowledge and behavioral change, while at the same time emphasizing the importance of the instructional design of the interventions [34]. Due to the stage in young people’s lives, which is associated with limited business acumen, among other things, dealing with financial issues at school seems abstract for many young people [35]. To close the perceived gap between economic theory and life practice for adolescents and young adults in upper secondary school, experience-based approaches such as GBL are suitable [32], because abstract economic models and processes can be run through and reflected upon in a failure culture, and proactive preparation for future decision-making processes can take place [7,12,36].

GBL is understood here as the instructional embedding of a serious game within a learning context. This includes an instructional introduction, game accompaniment, reflection prompts, and supplementary theory-based content transfer [37]. Unlike gamification, which is primarily aimed at increasing learning engagement [38], serious games are developed to achieve defined content-related learning objectives.

In general, GBL has an advantage in cognitive-learning outcomes, in which the promotion of motivational facets depends more on the instructional design of a GBL environment [4,39], which can also be confirmed to have a domain-specific perspective [12]. For example, there is evidence that GBL can change attitudes toward sustainability [40,41]. However, to this date there have been only a few studies on attitude development in the context of GBL or gamification [10]. For economics education, changes in attitude have so far been studied in relation to the application of serious games (attitude toward use) but not in relation to content [12].

### 2.3 Input–process–output model of serious game design

**Fig 1:**
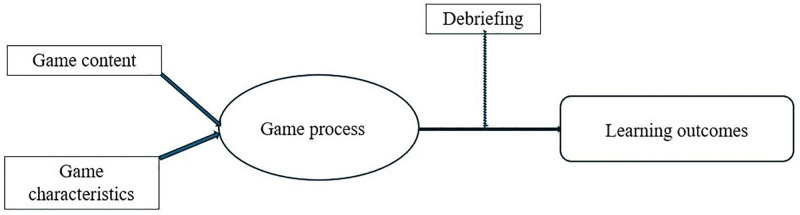
Input–process–output model of serious game design [15].

Based on the input–process–output model of serious game design by Garris, Ahlers and Driskell [15], learning objectives, in this case finance-related attitudes, are influenced by the input, the game process, and the accompanying debriefing, which functions as a moderator (Fig 1).

#### 2.1.3 Input in serious game design: Game mechanics.

One of the main building blocks of serious games is game mechanics, as they define game activities [42]. Among other things, game mechanics are systems of rules that players must follow and that therefore shape their interactions and behavior [43]. Empirically, the design of game mechanics is related to learning outcomes [44]. For example, game mechanics can influence situational interest, learning motivation, and cognitive learning outcomes [42,45–47]. Findings also suggest that specifically designed game mechanics can promote basic needs experience (BNE) in whole or at least in part [3,48].

If the designs of game mechanics reflect the intended learning objective, they can be called learning mechanics [45]. As serious games are designed to “engage players in meaningful learning activities*”* [45, p. 3], core game mechanics must be designed accordingly. If learning and game mechanics are not linked, it is possible that players may be motivated to play the game but not necessarily be motivated to learn [47]. In the case of promoting financial literacy through GBL, popular games such as Fantasy Football© or Cashflow101©, which follow a more linear and randomized approach in their game mechanics, already exist. As one main learning objective of financial literacy is to deal effectively with scarce resources, choosing a similiar game mechanics could lead to higher learning outcomes [37]. Therefore it can be assumed that a different approach that offers players a higher degree of autonomy and control is a recommended strategy [49].

In sum, the results of the design comparisons indicate the significant learning advantages of extended and thus strategic game mechanics [50]. By providing a wider range of possible actions and increased flexibility in terms of strategies, a greater sense of autonomy is expected when implementing strategic game mechanics [51]. Conversely, random game mechanics, such as rolling the dice, which restrict players or give them a sense of control, can reduce the feeling of autonomy and impair motivation [52,53]. Additionally, strategic game mechanics that offer increased challenges compared with more random game mechanics (e.g., dice) can increase the experience of competence [52]. Game mechanics that enable cooperation and interaction can increase the feeling of social inclusion [3]. These considerations suggest that strategic game mechanics may enhance autonomy and competence experiences, which in turn could foster more positive financial attitudes.

#### 2.1.4 Promoting basic needs experience during the game process.

The fact that Deci and Ryan’s self-determination theory (SDT) is the most commonly used theory to study GBL supports the assumption that autonomous motivation can be promoted through play [3,54]. In other words, the process of play itself is inherently perceived as motivating [55]. In the context of the empirically supported SDT, or its development into *basic psychological need theory*, autonomous motivation is promoted by the simultaneous satisfaction of three BNEs [55,56]: experiences of autonomy, competence, and relatedness are required not only to achieve higher performance outcomes but also for psychological growth and well-being [57].

Autonomy means that actions are based on one’s own interests and values and that one perceives oneself as the origin of behavior [56]. This can also mean that game rules are followed. However, in this case, the student cooperates out of a voluntary decision [58]. Therefore, to create an autonomy-supportive environment, providing attractive choices for students is fundamental. Relatedness refers to a perceived important and meaningful connection with other individuals or a group in which the focus is on the connection and not on an associated status [58]. Contrary to some assumptions, relatedness and autonomy are highly correlated. They occur together in the context of close relationships in which autonomy is to be understood as willingness or empowerment and not as individualism or non-reliance [59].

Competence refers to the experience of perceiving one’s own actions as effective and confident. This requires (learning) opportunities to practice and demonstrate already existing competencies and skills [57].

Meta-analyses have shown that intrinsic motivation for school activities decreases over the course of an individual’s school career, which can be attributed to a decline in psychological need satisfaction [60,61]. Therefore, creating a learning environment in which students’ psychological needs are met is also of interest in later school years. This is related to more autonomous learning motivation [57,59], which can lead to a positive attitude development toward school subjects or learning content [62–64].

#### 2.1.5 Debriefing as a moderator.

Debriefing through reflections prompts is seen as an important part of instructional support in GBL, as it can help with the processing and integration of information [65]. It can be defined as “the act of observing, inspecting, and contemplating (1) a belief or supposed form of knowledge, (2) the evidence that supports said belief or knowledge, and (3) the conclusions one draws from observing and inspecting one’s belief or knowledge” [66, p. 52]. As reflection prompts support students in connecting gameplay with learning, reflection prompts are offered in a game or by an instructor; both ways are related to positive learning motivation, while the effects vary by instructional design [66–68]. Recent empirical research suggests that reflection prompts designed to foster open-ended, free-form responses tend to be more effective in encouraging deeper reflection and comprehensive commentary than closed-ended prompts [69], But it is still unclear whether open or guided reflection prompts are more effective. An argument in favor of open-ended reflection prompts is that guided reflection can potentially limit opportunities for reflection and prevent learners from solving a problem independently, thus providing a higher degree of autonomy for problem-solving activities [67,70,71]. An argument in favor of guided reflection prompts is that learners’ metacognitive skills may not yet be well developed, and thus open reflection may be perceived as too difficult [71,72]. The learning context must also be taken into account: guided reflection is more likely to support more complex tasks [73]. Both can be effective and outperform no reflection prompts and lead to more in-depth results [74,75].

Therefore both game mechanics and reflection prompts must be aligned with the intended learning goals [45,76], which is also the case for serious games in general [77].

## 3 Research questions

Against this background, the following two research questions are addressed to study the influence of different game mechanics and reflection prompts on the game experience and finance-related attitude:

Research question 1:

How does the game experience in a serious game differ due to the variation in the core game mechanic and the design of reflection prompts?

**Fig 2:**
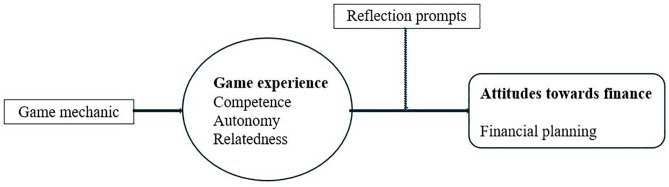
Working model.

Research question 2:

How do game mechanics influence the finance-related change in attitude under the mediation of game experience and the moderation of reflection prompts (Fig 2)?

## 4 Methodological approach

To study the influence of the design of game mechanics and reflection prompts, a previously developed game-based intervention program for upper secondary students was applied. The intervention program involved a GBL environment consisting of the serious game “Moonshot” and reflection prompts [37]. “Moonshot” is an in-house development created through an interdisciplinary collaboration. Experts from the fields of economics, education, pedagogy, and game development were involved in the two-year development process. The repeated piloting process was carried out with the involvement of stakeholders, such as teachers and students. For more details on the development process see [37].

### 4.1 Intervention program “Moonshot”

The intervention used *Moonshot*, an analog serious game for groups of three to five participants. Each participant selected one of several predefined “life dreams” (e.g., Olympic victory, influencer career, environmental reforestation) and pursued this objective under dynamic economic conditions.

Players managed at least one constrained resource – money – while simultaneously cooperating and competing with others. Decision options were structured across education, career, leisure, social engagement, and investment. Throughout play, participants documented their financial positions, including income, expenditure, assets, and liabilities. Randomized events (“destinies”) introduced financial risks, which could be mitigated through insurance. Progress toward a chosen life dream required combining earned income, investment activities, and risk management strategies. Game play was organized into two successive phases. Each phase was followed by reflection prompts. The integration of structured gameplay and reflection prompts enabled systematic engagement with all content areas of the OECD financial literacy competence framework.

### 4.2 Research design

**Fig 3:**
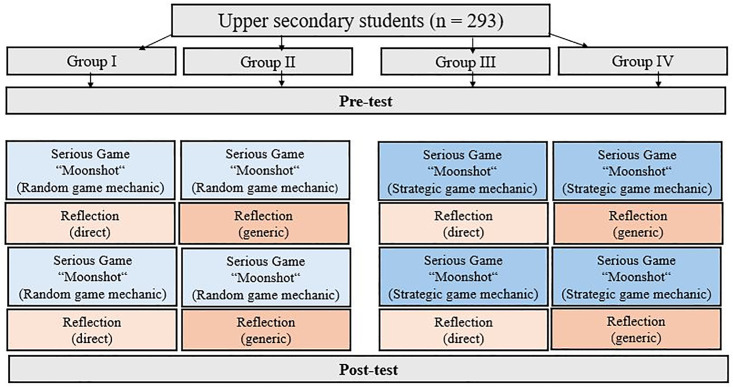
Intervention design.

A quasi-experimental approach with a 2 × 2 field design was chosen to address the research questions (Fig 3). A systematic variation was made in the intervention with regard to the game mechanics and reflection prompts. Groups I + II played the Moonshot game with a random game mechanic, while Groups III + IV played it with a strategic game mechanic. Between the two game phases, the players were asked to reflect on the game process. Groups I + III received direct prompts in the form of specific tasks, while Groups II + IV were generally asked to reflect on the previous game phase. The groups who received direct prompts had to evaluate in-game strategies, assess their transferability to personal financial contexts, and critically consider the broader implications of investment decisions.

The intervention took place from May to July 2022 in 17 different school classes at general secondary schools and vocational high schools in Germany. The implementation of the intervention was approved by the district council, and all students and—in the case of minors—their parents were informed of their rights regarding participation and data protection and gave their written consent. The Institutional Review Board (Ethics Committee of the University of Konstanz) confirmed the waiver of an ethics statement under the following reference number:: IRB24KN011–05/w.

Class sizes varied from 13 to 23 students. All classes were randomly assigned to the four groups while ensuring balanced representation of general education and vocational schools. It was implemented for one day (270 minutes) and consisted of instruction, as well as alternating phases of play and reflection. Developments in financial literacy and basic needs were measured using a digital questionnaire, which was deployed at five measurement points. The instruction was carried out by the same trained instructors to avoid confounding effects due to different teacher perspectives on the topic of finance [78].

### 4.3 Sample description

As described in the research design (Chapter 4.2), the sample consisted of four groups of students. The study was conducted with 293 upper secondary school students who were about to obtain their general qualification of university entrance. All students were enrolled in a general or vocational high school with or without economics as a main subject. Table 1 provides detailed information about the sample, separated by group affiliation. The groups were approximately the same size. In terms of gender, the majority were male students. The majority of the students were in a general education school with economics as their main subject and German as their native language. Age was typical for the grade. Slight differences were found in the distribution between the groups, which could be attributed to the fact that the allocation to the different groups took place at a class level (quasi-experimental research design).

**Table 1:** Sample description.

	Gender*		School Type**		Main subject***		Mother Tongue		Age	
	Female	Male	General	Vocational	Economics	Other	German	Other	M	SD
Group 1 (random, direct)	28 (37%)	47 (63%)	57 (78%)	16 (22%)	68 (91%)	7 (9%)	63 (82%)	14 (18%)	17.1	0.73
Group 2 (random, generic)	35 (47%)	39 (53%)	45 (63%)	27 (37%)	56 (76%)	18 (24%)	61 (82%)	13 (18%)	17.0	1.00
Group 3 (strategic, direct)	33 (44%)	42 (56%)	53 (73%)	20 (27%)	65 (88%)	9 (12%)	63 (84%)	12 (16%)	16.7	0.82
Group 4 (strategic, generic)	26 (39%)	41 (61%)	52 (80%)	13 (20%)	53 (82%)	12 (18%)	61 (91%)	6 (9%)	17.3	0.71
**Total**	**122 (42%)**	**169 (58%)**	**207 (73%)**	**76 (27%)**	**242 (84%)**	**46 (16%)**	**248 (85%)**	**45 (15%)**	**17.0**	**0.85**

*Notes:* M = Mean, SD = Standard deviation. Random/strategic describes the game mechanic, and direct/generic describes the reflection prompts (Chapter 4.2). *Two students declared “diverse” (not shown in the sample statistics). **10 students declared “both” or “other” (not shown in the sample statistics). ***Five students did not name a main subject.

### 4.4 Instrument

Table 2 presents an overview of the instruments used to measure the different variables of the theoretical model (Figs 1 and 2). The variables that had already been described in the sample statistics were not shown. The table also provides information on the scaling and reliability of the different variables. The reliability of all variables showed satisfying to good values [83].

**Table 2:** Overview of the instruments.

Variable	Items	Exemplary item	Scaling	Reliability**	Source
**Game experience**					
Competence	5	*During the game, my actions were important for my success.*	5-point Likert scale	.70	Adapted from [79]
Autonomy	5	*During the game, I was able to make challenging decisions on my own initiative.*	5-point Likert scale	.72	Adapted from [79]
Relatedness	6	*I received support during the game if I had any difficulties.*	5-point Likert scale	.79	Adapted from [79]
**Financial Literacy**					
Attitudes (financial planning)*	7	*I keep track of my money.*	4-point Likert scale	.82/.83	[80]
Self-efficacy*	5	*I lack confidence in my ability to manage my finances.*	4-point Likert scale	.73/.77	Translated from [81]
Motivation regarding finance*	6	*When I deal with financial issues, things excite me so much that I get completely involved.*	5-point Likert scale	.86/.83	[82]
Self-perceived financial knowledge*	1	*I generally consider my knowledge of finance to be...*	5-point Likert scale	- / -	Based on [82]

Notes: *The two values regarding reliability refer to T1 and T3 (before and after intervention). **Reliability is measured using Cronbach’s alpha.

### 4.5 Analysis strategy

To analyze the data, we used the statistical program Statistical Packages for the Social Sciences [84]. We also used the PROCESS macro version 4.2 by Hayes [85] to analyze the moderated mediations shown in Fig 2. All estimators were standardized. The scales were calculated using mean values based on classical test theory. Considering the students’ game experiences and reflections, the present study focuses on the second game circle because students at that time had already learned how to play and had played for a longer period of time.

#### Missing values.

There were almost no missing values on the item level. The highest number of missing values on the item level was 10 (about 3%). Accordingly, no systematic structure according to the item response could be assumed. In the case of missing values, listwise deletion was used.

#### Nested data.

As the students were nested in classes, structural differences between classes were assumed. To statistically consider these differences, the PROCESS macro offered robust standard errors [85] that were robust against violations of homoscedasticity.

## 5 Findings

### 5.1 Descriptive and bivariate statistics

Table 3 presents the descriptive statistics of the variables in the working model (Fig 2). Considering the descriptive results, there were little or no differences between the four groups before the intervention. By contrast, the game experience after the intervention strongly differed. Groups 3 and 4 had higher competence and autonomy experience and felt more socially integrated during game play than Groups 1 and 2. Comparing financial literacy before and after the intervention, most values slightly increased, with the exception of self-perceived knowledge in finance.

**Table 3:** Descriptive statistics (mean values and standard deviations).

Variable	Group 1 (random direct)	Group 2 (random generic)	Group 3 (strategic direct)	Group 4 (strategic generic)	Total
**Financial Literacy (before intervention)**					
Attitude toward financial planning	2.75 (0.57)	2.86 (0.57)	2.94 (0.46)	2.93 (0.53)	**2.87 (0.54)**
Motivation regarding finance	3.68 (0.63)	3.57 (0.69)	3.68 (0.75)	3.47 (0.75)	**3.60 (0.71)**
Self-efficacy	2.15 (0.52)	2.01 (0.62)	2.01 (0.52)	1.94 (0.56)	**2.03 (0.56)**
Self-perceived financial knowledge	3.19 (0.51)	3.12 (0.83)	3.08 (0.73)	3.04 (0.75)	**3.11 (0.77)**
**Game Experience**					
Competence	3.40 (0.94)	3.10 (0.90)	3.55 (0.77)	3.58 (0.81)	**3.41 (0.88)**
Autonomy	3.65 (0.91)	3.53 (0.89)	4.15 (0.72)	4.04 (0.83)	**3.84 (0.88)**
Relatedness	3.90 (0.87)	3.87 (0.87)	4.27 (0.68)	4.08 (0.99)	**4.03 (0.87)**
**Financial Literacy (after intervention)**					
Attitude toward financial planning	2.77 (0.51)	2.91 (0.52)	3.03 (0.45)	2.87 (0.58)	**2.89 (0.52)**
Motivation regarding finance	3.60 (0.68)	3.54 (0.73)	3.76 (0.75)	3.55 (0.62)	**3.62 (0.70)**
Self-efficacy	2.18 (0.51)	2.14 (0.54)	2.03 (0.48)	2.05 (0.66)	**2.10 (0.55)**
Self-perceived financial knowledge	2.89 (0.80)	2.94 (0.74)	2.86 (0.69)	2.92 (0.87)	**2.90 (0.77)**

Notes: Numbers in brackets represents standard deviations.

The differences between the four groups were analyzed in detail using a one-way multivariate analysis of variance (MANOVA) (see. Table 4). The results of the Levene test of the MANOVA showed homogeneity of the variance error for all dependent variables. By contrast, there was no homogeneity of covariances, as assessed by the Box test (p = 0.001). However, according to Verma [86] and Warner [87], the Box test in SPSS quickly becomes significant for larger groups; therefore, they suggest using a p-value of 0.001 instead of p = 0.05. In addition, SPSS uses statistics that are robust against violations of the equality of the covariance matrices of the dependent variables [88]. The post hoc statistics were not affected, which means that they were still interpretable [89]. The calculated one-way MANOVA showed a statistically significant difference between the four groups with regard to the combined dependent variables (*F*(33, 793) = 1.909, *p* < .001, partial η² = .072, Wilk’s Λ = .798).

**Table 4:** One-way multivariate analysis of variance (MANOVA).

Dependent Variable	F	df1	df2	p	η²
**Financial Literacy (before intervention)**					
Attitude toward financial planning	1.731	3	279	.161	.018
Motivation regarding finance	1.175	3	279	.320	.012
Self-efficacy	1.317	3	279	.269	.014
Self-perceived financial knowledge	0.567	3	279	.637	.006
**Game Experience**					
Competence	4.062	3	279	.008	.042
Autonomy	9.197	3	279	<.001	.090
Relatedness	3.171	3	279	.025	.033
**Financial Literacy (after intervention)**					
Attitude toward financial planning	3.198	3	279	.024	.033
Motivation regarding finance	1.511	3	279	.212	.016
Self-efficacy	1.719	3	279	.163	.018
Self-perceived financial knowledge	0.140	3	279	.936	.002

*Note:* Effect sizes are equal or similar when controlling for covariates like, e.g., gender or prior financial interest. The presented coefficients do not include any covariates. Only variables that are part of the theoretical model are considered.

No differences could be found for the other dimensions of financial literacy, self-efficacy, motivation and self-perceived financial knowledge. Thus, we conducted post hoc tests for competence experience, autonomy experience, relatedness, and attitude toward financial planning to observe the pairwise differences between the four groups. To calculate the robust effect sizes, we used the Tukey HSD adjustment, which is an appropriate adjustment if the homogeneity of the variance error is not violated; this is the case for these observed dependent variables.

#### Competence experience.

The Tukey HSD post hoc analysis on competence experience revealed a significant difference between Group 2 (random generic) and Group 3 (strategic direct), *p* = .013 (*M*_Diff_ = −0.44, 95% confidence interval (CI) [−0.81, −0.07]), as well as in Group 4 (strategic generic), *p* = .017 (*M*_Diff_ = −0.44, 95% CI [−0.83, −0.06]). Thus, groups with a strategic game mechanic perceived a higher competence experience during game play than groups with a random game mechanic and generic reflection. No further differences in the reflection prompts were identified.

#### Autonomy experience.

The Tukey HSD post hoc analysis on autonomy experience revealed a significant difference between Group 1 (random direct) and Group 3 (strategic direct), *p* = .002 (*M*_Diff_ = −0.48, 95% CI [−0.83, −0.13]), as well as in Group 4 (strategic generic), *p* = .024 (*M*_Diff_ = −0.40, 95% CI [−0.77, −0.04]). Significant differences were found between Group 2 (random generic) and Group 3 (strategic direct), *p* < .001 (*M*_Diff_ = −0.60, 95% CI [−0.96, −0.25]), as well as in Group 4 (strategic generic), *p* = .002 (*M*_Diff_ = −0.52, 95% CI [−0.89, −0.15]). Thus, groups with a strategic game mechanic perceived a higher autonomy experience during game play than the groups with a random game mechanic. No differences in the reflection prompts were identified.

#### Relatedness.

The Tukey HSD post hoc analysis on relatedness revealed a significant difference between Group 2 (random generic) and Group 3 (strategic direct), *p* = .040 (*M*_Diff_ = −0.37, 95% CI [−0.73, −0.01]). Thus, groups with a strategic game mechanic and direct reflection perceived higher relatedness during game play than groups with a random game mechanic and generic reflection.

In sum, a random game mechanic combined with a generic reflection leads to basic needs being satisfied the least. By contrast, a strategic game mechanic leads to a high satisfaction of competence and autonomy experience. Combined with direct reflection, relatedness is also satisfied more strongly.

#### Attitude toward financial planning.

The only group-specific differences regarding financial literacy were observed for attitudes toward financial planning. The Tukey HSD post hoc analysis revealed a significant difference between Group 1 (random direct) and Group 3 (strategic direct), *p* = .013 (*M*_Diff_ = −0.26, 95% CI [−0.48, −0.04]). Thus, groups with a strategic game mechanic and direct reflection showed a more positive attitude toward financial planning than groups with a random game mechanic and generic reflection.

Based on these bivariate results, the theoretical model (Figs 1 and 2) is tested in the following section. The mediating effect of game experiences and the moderating effect of reflection prompts are observed in more detail. As the only difference was found for attitude after intervention, the prediction of financial literacy was restricted to this dependent variable.

### Prediction of financial literacy by game mechanics and reflection prompts

**Fig 4:**
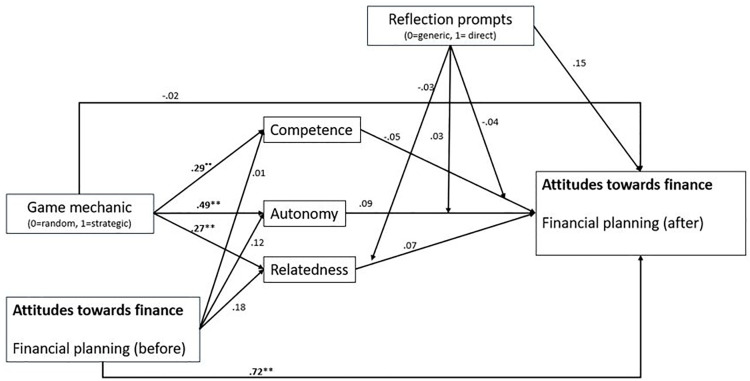
Path coefficients of the reflection prompts represent the interaction effects of the reflection prompts with game experiences (competence, autonomy, and relatedness).

Fig 4 presents the different path coefficients of the direct effects based on the theoretical assumption.

The results show that game mechanics strongly affected the game experiences of students. By contrast, these game experiences did not influence financial literacy (i.e., attitudes toward finance).

Considering the indirect effects of game mechanics on attitudes toward finance, a small mediation effect of autonomy could be assumed to be moderated by reflection. Thus, students with a direct reflection benefitted from a strategic game mechanic more strongly, as zero was not included in the 95% CI (bootstrap with 20,000 bootstrap samples, 95% CI [0.001, 0.103]). In addition, the mediating effects of relatedness were positively moderated by the reflection prompts. In this regard, students with both direct and indirect reflections benefitted more strongly from a strategic game mechanic (bootstrap with 20,000 bootstrap samples, 95% CI [0.001, 0.046] for direct reflection and 95% CI [0.004, 0.062] for generic reflection). The remaining indirect effects were not significant.

## 6 Discussion

The primary objective of this study was to identify effective instructional design features of serious games in the context of school-based education, with a particular focus on promoting financial literacy. We therefore compared two distinct game mechanics: one commonly applied in existing financial games and another defined by its stronger alignment with the promotion of financial literacy. Each mechanic was paired with one of two reflection prompts previously shown to be effective. Using multivariate analysis of variance and path modeling, we assessed the impact of these interventions on the gaming experience – conceptualized as BNE – as well as on finance-related attitudes, specifically attitudes toward financial planning. To guide our analysis and interpretation, we drew on an adapted input–process–output framework [15], which links game elements, user experience, and learning outcomes in educational contexts.

Regarding the first research question, the one-way MANOVA indicated a statistically significant overall effect of the manipulations on the combined dependent variables (Table 4). Tukey HSD post hoc tests revealed that the combination of a strategic game mechanic with guided reflection prompts produced a significant increase in BNE, particularly concerning autonomy. A significant group difference was also observed for finance-related attitude, though this effect was limited to the comparison between Group I and Group IV. The enhanced BNE, especially autonomy, in the combined condition suggests that strategic mechanics and guided reflection act synergistically rather than independently. This pattern indicates that strategic mechanics impose greater cognitive complexity, which in turn makes guided reflection necessary to support effective processing and learning consolidation. The superiority of this combined approach are in line with recent findings [90], who demonstrated the benefits of inquiry-based mechanics for learning motivation and knowledge acquisition. While they focused on inquiry-based design, our results suggest a parallel dynamic for strategic mechanics: in both cases, challenging game mechanics require complementary reflection prompts. Our findings also broaden current discourse on inclusive game-based learning [10,32], indicating that the integration of strategic mechanics with guided reflection may represent an effective design strategy for fostering BNE across diverse learner groups.

At the same time, the analysis provides only limited evidence that positive gaming experiences consistently transfer into content-related outcomes. This became clear when addressing the second research question. The subsequent path model with moderated mediations confirmed the influence of game mechanics on BNE but did not account for the group-related difference in finance-related attitudes under the moderation of reflection prompts (Fig. 4). These findings suggest that trait-related influences can be fostered within a comparatively brief intervention, whereas effects on state variables – specifically attitudes – remain difficult to detect. Three explanations appear most plausible. First, the short duration of the intervention may not have been sufficient to trigger attitudinal change [6,32]. Second, a closer alignment between the measures of gaming experience (as they relate to finance-related attitudes) and the assessed learning outcomes might have lead to stronger effects. Third, given our reliance on manifest variables, effect sizes may have been underestimated [91]. Despite these limitations and the theoretically anticipated weak effects for trait-related variables, we opted for a comprehensive modeling approach in order to map the full theoretical framework. This provides an essential foundation for subsequent studies that can employ more robust measurement approaches and longer intervention periods.

## 7 Limitations and future directions

Answering the questions in the context of the quasi-experimental design must be discussed against the background of some limitations. The fact that these group-related differences in attitude cannot be explained here is also due to the overall low effect size. Even if the group-related differences of the one-way MANOVA could provide an initial indication, the influence of the instructional design of the game mechanics and reflection prompts on finance-related attitude could probably not be confirmed due to the relatively short interval between the first and last points of measurement (270 minutes). So it can be assumed that the effects of the variations described will only become apparent over a longer period of time. The small effects of attitude change toward financial planning are consistent with cross-domain reviews, which have a mean value of d = 0.22 for one-time interventions [28]. Sensitivity power-analysis with G*Power for MANOVA (global effects) showed that with the present sample, it is possible to detect significant effects of η² = 0.038 (α = 0.05; β = 0.90; number of groups = 4) [92,93]. In addition, the calculated models were all based on a manifest measurement of the variables because the latent models with three mediators, with each mediator moderated by reflection, led to consistency problems of the model. This is again due to the relatively small sample or groups. Considering this, it must be assumed that the calculated estimators are probably underestimated because of residual variances that could not be excluded. This could be another reason for the overall small effect sizes and the lack of significance of the path coefficients. Thus, replication studies with a bigger sample are highly recommended, to ensure that even minor effects can be reliably identified.

Another limitation must be seen in the specific context of the study that is limited to a small sample with just few school classes at upper secondary education where most students have chosen economics as their main subject in school. Thus, no class level variables are considered, such as teacher instructions or classroom interactions, which limits the internal validity of the study. Furthermore, it must be questioned, to which extent these results can be transferred to other contexts (external validity), such as lower secondary education, vocational education, or also higher education. Thus, further replication studies with different contexts are highly recommended.

With regard to the selected measurement instruments, it can also be assumed that more recent approaches can more clearly depict interdependencies. These include, for example, the measurement of BNEs during the game. Recent approaches to serious games also consider not just need satisfaction but also need frustration [94]. In addition to the BNE and utility value, the extent to which other motivators, such as entertainment, also influence the effectiveness of serious games should be examined [95]. Supplementary validation can be provided by process mining approaches using log files as a basis for validation [96].

Moreover, the inclusion of additional control variables can reveal possible confounding effects. The study did not determine how the students actually made use of the applied game mechanics. Through observations during gameplay, it was obvious that there were different approaches to making use of strategic game mechanics. Therefore, it could be necessary to control their approach and focus on what prerequisites influence the use of game mechanics [44]. There are hints that the probability of switching from static to dynamic game mechanics over time is increasing [44].

Against this background, it can also be assumed that Garris et al.‘s [15] model for explaining the acquisition of skills in serious games falls short of the mark and that subsequent studies should further develop this basis empirically.

## 8 Implications

Despite these limitations, the findings generated by the study have substantial implications for (1) research and (2) practice: (1) Based on learning and model theory, it was empirically shown that a slight change in the game mechanics could significantly influence BNE during game play. This is particularly evident when strategic game mechanics are combined with direct reflection prompts. In-depth analyses on a qualitative level of students’ reflections are necessary to understand how these are related to skill and knowledge acquisition [76]. This study is the first to investigate the influence of two combined game design elements on game experience, which is a key predictor of attitude change, finance-related attitudes, and behavior change [6]. However, this requires further intervention studies over a longer period of time and with a larger sample size to be able to map process-related developments even more comprehensively using structural equation models to examine how financial education influences attitudes toward finance and what kind of change could serve financial well-being. It can be assumed that the identified changes in the gaming experience can lead to changes in finance-related attitude over a longer period of time and that these changes can influence behavior.

This study establishes an initial foundation that may be systematically extended to domains beyond finance: To the best of our knowledge, this is the first study to show the effects of different game mechanics and reflection prompts of GBL based on an experimential pretest-posttest design. Such an empirical analysis based on a solid theoretical foundation is generally rare [97] and provides relevant contributions applicable to a broad range of educational contexts. This is especially true for BNE, since their importance can be confirmed across different cultural backgrounds, while promoting a positive attitude towards planning may require greater cultural and socio-economic sensitivity when adapting interventions. This also applies to game mechanics – while strategic game mechanics may be effective across domains due to their explorative and strategic characteristics, other domains may require different, more content-aligned game mechanics. For a practical overview, see [46].

(2) Practical implications for the development and application of serious games in economics education and beyond can also be derived. For example, the motivational application of serious games depends on game mechanics that structure game actions. These include game mechanics that are associated with greater strategic freedom for players, which can be considered independent of indented learning goals by providing a more explorative approach towards learning. A further step toward increasing the experience of autonomy and competence could be the addition of adaptive features [98]. In the case of finance-related serious games, this step could be, for example, the development of personal income or wealth targets. It is important to take this into account, as media comparisons show that the use of serious games does not per se lead to greater learning motivation compared with other media, especially in secondary and tertiary education (see [12,39]). Debriefing that uses well-structured prompts to encourage reflection on players’ experiences and a transfer to the reality of their lives needs to be also considered. Simply providing strategic freedom without offering the necessary support to utilize it does not automatically lead to greater competence or autonomy. This may also apply to other simulation-based learning media like case studies. And it cannot be assumed that simply asking teenagers and young adults to reflect is sufficient, especially with accompanying challenging serious games. Since designing guided reflection prompts is demanding, AI chatbots may offer effective support for developing individualized and targeted reflection prompts [99].

### Conclusion

This study demonstrates that integrating strategic game mechanics with guided reflection supports the enhancement of BNE in financial education contexts. The results indicate that thoughtful design – where cognitively challenging gameplay is paired with structured reflection – can improve student engagement and foundational learning experiences. Nevertheless, the impact on finance-related attitudes appeared limited in the short term, likely reflecting both the brevity of the intervention and method-related constraints. These findings underscore the need for future research employing extended interventions and advanced analytic models to clarify how serious games might shape attitudinal and behavioral change over time. Overall, the study highlights that serious games in education are most effective when carefully designed to balance engaging mechanics with structured opportunities for reflection and transfer. Therefore, the combination of strategic freedom during play and structured guidance during debriefing offers an opportunity to apply serious games effectively in the classroom.

## Supporting information

S1 File Inclusivity-in-global-research-questionnaire. (DOCX)
